# Paralysis—Manganum

**Published:** 1887-04

**Authors:** E. W. Berridge

**Affiliations:** London, England


### PARALYSIS—MANGANUM.

E. W. Berridge, M. D., London, England.

In reply to Dr. Gee, the peculiar paralysis which I could only find under *Manganum* was *the inclination to run forward if he tried to walk.* The difference of direction in the paralysis of *Mang.* and *Merc.* was noted by Dr. Cooper, who seems to have been more observant than allopaths usually are. C. Lippe's Repertory also gives *Hydrocy.-acid* under paralysis, first of lower limbs, then upper. My thanks are due to Dr. Gee for calling attention to *Conium.* Dr. C. C. Smith's characteristic of *Stram.*, "vomiting from exposure to bright light," resembles one of *Opium*, which I found recorded in a case of poisoning, "vomiting from gas-light." Dr. Boyce has been misinformed as to Bœnninghausen's directions for the treatment of croup. He will find the correct version in *American Homœopathic Review*, vol. 2, p. 561. Besides, these directions were simply the best that could be given to the public in the absence of a homœopathic physician.

